# Evidence-Based Approaches to Remedy and Also to Prevent Abuse of Community-Dwelling Older Persons

**DOI:** 10.1155/2011/861484

**Published:** 2011-08-14

**Authors:** Donna M. Wilson, Sandra E. Ratajewicz, Charl Els, Mary A. Asirifi

**Affiliations:** ^1^Faculty of Nursing, University of Alberta, Edmonton, AB, Canada T6G 2G3; ^2^School of Public Health, Faculty of Medicine and Dentistry, University of Alberta, Edmonton, AB, Canada T6G 2R7

## Abstract

Elder abuse is a global issue, with an estimated 4–10% of older persons in Canada abused each year. Although Canadian legislation has been created to prevent and punish the abuse of older persons living in nursing homes and other care facilities, community-dwelling older persons are at greater risk of abuse. This paper highlights the importance of evidence-based actions targeted at three determinants of health: (a) personal health practices and coping skills, (b) social support networks, and (c) social environments. Two research studies are profiled as case studies that illustrate the ready possibility and value of two specific types of actions on community-based older-person abuse. This paper argues for the immediate and widespread adoption of these evidence-based measures and for additional empirical evidence to guide the correction of underreporting of abuse, raise awareness of its serious nature, and increase options to not only stop it but ultimately prevent it.

## 1. Introduction

The World Health Organization defines elder abuse as an intentional or unintentional single act or multiple acts and/or omissions that result in distress or harm to older adults, with this harm being “physical, verbal, psychological/emotional, sexual, and/or financial in nature” [1]. Between 4% and 10% of older Canadians each year are estimated as experiencing abuse of one form or multiple forms, with similar rates reported in some other countries [2–6]. It is challenging to define the exact parameters of this vulnerable subpopulation, as there continues to be much confusion about what actually constitutes older-people abuse [7]. Limitations in identification and reporting systems also make it very difficult to estimate the exact incidence or prevalence of elder abuse [4]. However, existing evidence indicates abused older persons are more likely female, cognitively impaired, in poor physical health, and dependent on other persons [8].

Abuse has short- and long-term effects on the person's physical, mental, emotional, and spiritual well-being [9]. Afflicted persons first have to cope with the pain, stress, and other direct impacts of the abuse. Illnesses are often triggered by abuse; such as depression, hypertension, stroke, and heart attacks [10], with these illnesses contributing to the burden of abuse. As such, elder abuse is a serious problem that needs to be detected and remedied quickly and effectively and preferably prevented. As the populations in both developed and developing countries are rapidly aging now [11, 12], the number of people at risk of elder abuse is rapidly increasing. Given these considerations, there is a dire need for immediate action on elder abuse.

## 2. The Population Health Promotion Model

Although concern in Canada has focused largely on the abuse of older persons in nursing homes and other care facilities [13], community-dwelling seniors have a much higher risk of abuse. In large part, this risk is simply because 90–95% of older adults reside in their own homes or apartments [14], places with little public oversight. There is also a dearth of services or programs designed specifically to detect, address, and prevent the abuse of community-dwelling older persons [6, 8]. Universal broad-based interventions and also targeted interventions to prevent and address elder abuse are needed, and these ideally should be based on health promotion frameworks, such as the Population Health Promotion Model (PHPM) [15], a widely referenced and used model.

The PHPM is a three-dimensional framework to assist in the determination of what, how, and with whom action should be taken to maintain or improve health [15]. This model is encouraging of actions that reduce deficiencies and/or enhance strengths in relation to 5 health promotion strategies: (a) strengthening community action, (b) building healthy public policy, (c) creating supportive environments, (d) developing personal skills, and (e) reorienting healthcare services to be health promotive [15]. The actions taken should also be aimed at 1 or more of the 12 determinants of health (as listed in Table 1). Finally, research evidence should inform about the actions selected for implementation.

In the case of elder abuse, interventions to prevent and to address abuse once detected should be based on evidence to ensure that relevant issues are recognized and the most effective actions are taken [15]. There is only a limited body of intervention research on elder abuse, however. Two studies among others illustrate both the need for and value of evidence-based interventions for community-dwelling older persons at risk of abuse or experiencing abuse [8, 17]. These research investigations provide case study illustrations of the ready possibility and value of actions directed at community-based older-person abuse.

### 2.1. Family Care Conferences

A research investigation was undertaken by Holkup, Salois, Tripp-Reimer, and Weinert to evaluate a community-based intervention designed to end older-people abuse, identified as the “Family Care Conference” [17]. Although family group therapy, family meetings to discuss the care of ill family members, and family-based approaches to improve the health of an individual or the family as a whole have been employed for many years, family group conferences aimed at stopping the abuse of an older family member are a relatively new and as yet largely untested approach [18–22]. For instance, although Tapper reports family care conferences have been used in New Zealand since the 1990s for child welfare and protection situations, as they resulted in decreased domestic violence, they are only now being used in New Zealand for cases of detected elder abuse [18]. Early positive outcomes of this new elder abuse program (i.e., the “Bluebird Project”) are already evident, however, as the abuse is stopped because the vulnerable adult is safeguarded. Perhaps the greatest indicator of success with the Bluebird Project is that the families support this approach [18]. Family members report feeling respected and valued because they are listened to by everyone attending their family care conference [18]. A Canadian study on the value of family conferences for addressing child abuse similarly revealed that family unity and family member safety are clear outcomes, as these conferences initiated open and constructive dialogue among family members about the problem and also fostered cooperation among family members [20]. One additional positive outcome is their effect on healthcare providers; through their involvement in these conferences, they came to realize the value of whole-family involvement and input, and begin to focus more on families and less on an individual family member in other care situations [20].

In Holkup et al.'s intervention study, each Family Care Conference was set up with a facilitator to govern a meeting comprised of the abused older person, their family members, additional community members (if deemed relevant by the affected older person), and a selection of healthcare and/or social service professionals [17]. At each conference, the expectation was that concerns would be voiced equally by all group members, endorsing the principle that both the abused and the alleged perpetrator(s) need to have their concerns and issues openly identified. Through the critical input of healthcare and/or social service professionals, more objective viewpoints could also become known. After a frank discussion, the family as a whole was then encouraged to develop a plan of action, with this plan to include how it would be implemented and evaluated. Of the 22 Family Care Conferences studied, 10 yielded clear benefits. These benefits were largely believed to occur because family members appreciated the constructive approach taken.

As such, Holkup et al.'s [17] study did not reveal an outstandingly successful approach for addressing elder abuse, a conclusion that emphasizes the fact that older-person abuse is typically complex and multifaceted, and thus difficult to address. Older-person abuse can also be longstanding and thus refractory, as the abuse may have its origins in family dynamics of many-year duration [23]. Holkup et al.'s study is relevant and important, however, for highlighting one practical and potentially useful intervention that could be employed in any community and any abuse case.

Holkup et al.'s [17] study is also notable for emphasizing 3 health determinants as being particularly relevant for conceptualizing and preventing elder abuse: (a) social support networks, (b) social environments, and (c) personal health practices and coping skills. These health determinants are highly interconnected, as illustrated by the following. Abused older persons tend to be those who are socially isolated, often because of being house bound and therefore reliant on others for meeting basic needs, such as transportation and perhaps also ambulating within and outside the home [23]. Inadequate social support networks would also be a result of having lost spouses or other supportive family members and close friends over time. In addition, many have age- or illness-based cognitive and/or physical impairments that increase their risk of abuse, but these also require them to develop new personal health practices and coping skills. Older persons at risk of elder abuse thus typically have reduced physical and social capacity to make new friends, and those who are abused typically lack confidence because of the abuse and they often experience embarrassment about it [23]. Older adults with such limitations are less likely to report abuse, and at the same time, they also have diminished means or capacity to stop the abuse by themselves [16].

If championed by a nurse, another healthcare professional, and/or community leader, Family Care Conferences (or their equivalent) could be initiated at low cost in any community. The lessons gained through Holkup et al.'s study [17] and other studies focused on whole family approaches [18–22] would support this initiation. In turn, the knowledge and skills gained from each Family Care Conference would enhance and strengthen this approach for stopping elder abuse. This approach has considerable promise, as Family Care Conferences have the potential to have a direct beneficial effect on the abused person and their abusers. They could also serve to heighten awareness of older-people abuse at all levels of society (i.e., individual, family, local community, and broader society). At the very least, these Conferences could stop the abuse, while enhancing the personal capacity and support network of the abused older person to ensure that the abuse does not resume or that continued abuse does not go unreported. Family members could also gain by being provided opportunities to learn how to stop and how to prevent ongoing abuse. As such, Family Care Conferences illustrate important primary and secondary health promotion considerations [16].

In conclusion, Family Care Conferences could be an effective way to strengthen individual and community action against older-person abuse. Community empowerment is critical, as communities need to be more willing and capable of recognizing elder abuse as a major issue in need of direct and immediate action. Communities that are empowered will make decisions, plan strategies, and implement these strategies for improved health outcomes for abused and potentially abused seniors. Holkup et al.'s [17] family conference approach supports the view that communities should take ownership of abuse to improve the well-being of community-dwelling older people.

 Open and direct action such as this is recommended by the *Canadian Network for the Prevention of Elder Abuse *[9], a voluntary organization that believes solutions are best derived through partnerships, as joint efforts ultimately build community capacity [24].

### 2.2. Interdisciplinary Action

Barker and Himchak's study also holds much promise for building community capacity to remedy older-person abuse [8]. Barker and Himchak surveyed 126 older abuse victims in England who lived alone, examining if these respondents sought help, which services they utilized, and which services were most beneficial to them. Their study revealed many did not utilize any services, as most felt ashamed of being abused and they were unwilling to have their abuser (often a family member) punished. Their responses further revealed that they believed few abuse reduction or prevention services were available and that there was limited access to these scarce services. The most effective intervention, as endorsed by those who had used an intervention, was an interdisciplinary agency where they had access to a wide range of professionals. Together, these professionals were considered capable of understanding and addressing their abuse from multiple viewpoints.

Other studies have also identified interdisciplinary teams as the hallmark of abuse treatment programs [25–31], including the Family Care Conference study described above [17]. A collective interdisciplinary approach is understandable, as abused older persons typically have many interrelated issues affecting them, in part because as they are unlikely to suffer from only one form of abuse. They could be suffering at the same time from neglect, physical abuse, emotional abuse, and financial abuse; while also having to cope with an age-based or illness-based decline in cognitive and/or physical health. It is therefore a logical deduction to suggest that an interdisciplinary approach is most often needed, not only to sufficiently understand the phenomenon, but also to be more successful at stopping elder abuse and preventing elder abuse.

Community health nurses, other healthcare professionals, and/or community leaders could use the research evidence on interdisciplinary abuse teams to advocate for teams to be situated in primary care clinics and hospital emergency departments, as these are the two most common first-contact points with the healthcare system [7]. However, this team approach would represent a major shift from the current illness-oriented and single-provider focus in most healthcare systems [1]. Once established, these teams would need to be trained to employ a collaborative process to meet each abused individual's needs both immediately and over the longer term [8]. Another community-based abuse reduction option was identified by Reingold, who recommended a mobile outreach van to take interdisciplinary abuse services to older persons who are unable to travel [25].

One advantage of interdisciplinary approaches is that teams and not individuals would share the responsibility of helping vulnerable abused or at-risk older persons, and this collective approach would also be more holistic [26]. It is important to recognize that no single discipline is capable of sufficiently understanding and resolving all causes or consequences of abuse; interdisciplinary approaches therefore ensure that multiple aspects of each abused person's well-being are assessed and addressed [23, 25–27]. Multiple risk factors or contributors to abuse are also more likely to be considered by teams—such as social isolation, emotional and physical dependency, low income and education, housing issues, financial improprieties, and perhaps even substance abuse [8]. Ultimately, group-based interventions should be more successful at stopping abuse once detected, and also more successful at reducing the incidence of older-person abuse through increased professional awareness of it, and through fostering cooperation across professional and community groups to stop it [25].

Perhaps above all else, interdisciplinary abuse teams could advocate for legislation, making it mandatory for healthcare professionals and other persons to report suspected cases of community-based older-person abuse. Currently, mandatory reporting of abused or potentially abused children and animals is in place in many jurisdictions. In some regions, such as the province of Alberta, Canada, reporting suspected cases of abuse of older persons in nursing homes and other publicly funded facilities has been required since 1998 [13]. To date, no perpetrator has ever been prosecuted under this act, which suggests the Act may not be in fact serving its purpose. The authors believe this act and others like it should be extended to include the most likely place where older-person abuse occurs—the community.

## 3. Conclusion

Elder abuse is an underreported and thus minimally recognized issue affecting a significant proportion of older persons. Existing information suggests there are insufficient efforts in Canada and elsewhere to detect it, as routine and accurate recording of it is lacking. Consequently, few corrective and prevention services exist. Despite these challenges, research studies clearly show it is possible to successfully remedy older-person abuse. Two promising evidence-based approaches were highlighted as case studies for consideration: Family Care Conferences and Interdisciplinary Abuse Teams. Although further research is required to refine these interventions and test others, research translation is required now for immediate action on elderly abuse. Many abused seniors currently suffer in silence and many more could suffer in the years ahead as population aging rapidly increases the number of at-risk persons worldwide [11, 12]. Elder abuse clearly can be stopped once it has started, and it can also be prevented.

## Figures and Tables

**Table 1:** The twelve determinants of health.

(1) Income and social status
(2) Social support networks
(3) Education and literacy
(4) Employment/working
(5) Social environments
(6) Physical environments
(7) Personal health practices and coping skills
(8) Healthy child development
(9) Biology and genetic endowment
(10) Conditions health services
(11) Gender
(12) Culture

*Source: [16].
